# Relationship between sarcopenia and fatty liver in middle-aged and elderly patients with type 2 diabetes mellitus

**DOI:** 10.1186/s13018-024-04717-9

**Published:** 2024-04-20

**Authors:** Li Quan, Fang Zhang, Jing Xu, Fei Wang, Yong Fan

**Affiliations:** State Key Laboratory of Pathogenesis, Prevention and Treatment of High Incidence Diseases in Central Asia, department of endocrinology, The first Affiliated Hospital of Xinjiang Medical University, No. 137 of Liyushannan Street, Xinshi District, Urumqi, 830054 China

**Keywords:** Non-alcoholic fatty liver disease, Sarcopenia, Type 2 diabetes mellitus

## Abstract

**Objective:**

In this study, we investigated the relationship between sarcopenia and fatty liver in middle-aged and elderly patients diagnosed with type 2 diabetes mellitus (T2DM) to provide a theoretical foundation for the prevention and treatment of sarcopenia.

**Methods:**

A total of 282 patients diagnosed with T2DM aged 50 and older and were admitted to the Endocrinology Department of Xin Medical University First Affiliated Hospital between December 2021 and February 2023, were selected. Body mass index (BMI), and limb and trunk muscle mass of the patients were measured, and data were collected. Patients were grouped based on the sarcopenia diagnostic criteria. All study participants underwent the same physical examinations and laboratory tests. The relationship between the onset of sarcopenia and fatty liver in middle-aged and elderly patients diagnosed with T2DM was then investigated using statistical analysis.

**Results:**

Comparing the sarcopenia group to the non-sarcopenia group revealed statistically significant variations in gender, BMI, fatty liver prevalence rate, uric acid (UA), alanine aminotransferase (ALT), blood glucose, blood lipid associated indicators, and limb skeletal muscle content. There were, however, no statistically significant differences in age, disease duration, hypertension, smoking, or alcohol intake. There was a positive correlation between BMI, UA, fasting c-peptide, and Appendicular Skeletal Muscle Index (ASMI). Higher levels of BMI, ASMI, and UA were identified as protective variables against sarcopenia by multifactorial logistic regression analysis.

**Conclusion:**

Higher levels of BMI, ASMI, and UA can greatly reduce skeletal muscle atrophy in patients with T2DM. Patients with a fatty liver may be less vulnerable to sarcopenia. There is little evidence, however, that a fatty liver works as a preventive factor against sarcopenia.

## Introduction

Diabetes is a chronic systemic disease. Between 2013 and 2018, the prevalence of diagnosed diabetes among adults in China increased significantly [1]. Diabetes is widely assumed to be caused by a reduction in the insulin sensitivity of various tissues and organs throughout the body, or by a decrease in endogenous insulin secretion [2]. The number of patients with T2DM is growing annually, making it a global public health concern. Diabetes affects around 38.1% of the middle-aged and elderly population, according to research [3]. 

Prolonged hyperglycemia in diabetes often lead to an increased incidence of fatty liver disease [4]. Non-alcoholic fatty liver disease (NAFLD) is a form of metabolic liver disease that is closely linked to insulin resistance and genetics [5, 6]. In China, it has become the leading cause of increasingly severe chronic liver disease [7]. A meta-analysis of 1 832,125 patients identified the prevalence of Non-alcoholic fatty liver disease (NAFLD) in T2DM as 65.04%, respectively [8]. 

Insulin resistance in T2DM may exacerbate the condition of hyperglycemia, leading to a decrease in skeletal muscle mass and function, which is associated with sarcopenia, also known as muscle wasting [9]. Sarcopenia is a chronic metabolic disease characterized by a reduction in muscular strength and mass [10]. It is a disorder that progressively worsens and is frequently associated with other chronic diseases, such as T2DM, metabolic syndrome, cardiovascular disease, and other chronic conditions [11]. The prevalence of sarcopenia in the community ranged from 5.5 to 25.7% when using the AWGS2014 diagnostic criteria, with males having a higher prevalence than females. The prevalence of sarcopenia in Asia ranged from 7.3 to 12.0% [12]. Studies have revealed that muscle mass declines as glycated hemoglobin (HbA1c) levels rise, indicating a link between the beginning of sarcopenia and T2DM [13]. One study found that people with diabetes were 73% more likely to develop sarcopenia [14]. Additionally, patients with sarcopenia have an increased risk of developing osteoporosis [15]. Consequently, middle-aged people and the elderly with sarcopenia are more prone to falls and fractures, resulting in a significant reduction in their quality of life. Some may even become dependent on others for everyday activities, and sarcopenia becomes a major contributor to disability and mortality [16, 17]. 

There is currently no conclusive evidence indicating the link between sarcopenia and fatty liver in patients with diabetes. The purpose of this study is to examine the relationship between muscle wasting and fatty liver in middle-aged individuals and the elderly with T2DM, to provide a theoretical basis for the prevention and treatment of sarcopenia.

**Research Content and Methods**.

### Study participants

Participants in this study were all patients diagnosed with T2DM admitted to the Endocrinology Department of Xin Medical University First Affiliated Hospital between December 2021 and February 2023, aged 50 years or older. The participants were then divided into the sarcopenia group/case group and the non-sarcopenia group/control group based on the 2019 diagnostic criteria for muscle wasting developed by the Asian Working Group for Sarcopenia [12]. This study was approved by the local ethics (approval no. K202311021) committee on November 10th, 2021. All participants provided signed written informed consent.

### Inclusion criteria

1. Aged 50 years and above, both males and females.
2. Meet the diagnostic criteria for diabetes published in 1999 by the World Health Organization (WHO) [15].
3. Participants free of mental diseases and competent to undertake the essential tasks of the study.
4. Those who comprehended the experiment and agreed to participate.

### Exclusion Criteria

1. Below the age of 50 years.
2. Patients with Type 1 diabetes or other special types of diabetes.
3. Patients in the acute phase of any disease or with severe chronic organ failure.
4. Damage to the liver resulting from excessive alcohol intake, viral infections, drug-induced injuries, and autoimmune disorders.
5. Long-term and current use of drugs that change bone density, signs of bone metabolism, and internal hormone levels.
6. Patients suffering from degenerative diseases such as tuberculosis and malignant tumors.

## Content and methods

### Data collection

#### General data

Name, gender, age, ethnicity, diabetes disease duration, smoking history, alcohol consumption history, history of other chronic diseases (such as hypertension, coronary artery disease (CAD), and history of thyroid disease), family history, height, weight, and so on will be recorded for all study participants. All these details were meticulously recorded.

## Laboratory examination

After fasting overnight for at least 8 h, blood samples were collected from all participants in the morning following their admission. Blood samples were collected by the nurses from the endocrinology department. Tests included fasting blood glucose (FBG), postprandial blood glucose (PBG), fasting c-peptide (FCP), HbA1c, alanine aminotransferase (ALT), aspartate aminotransferase (AST), creatinine (Cr), uric acid (UA), albumin (Alb), triglycerides (TG), total cholesterol (TC), high-density lipoprotein cholesterol (HDL-C), low-density lipoprotein cholesterol (LDL-C), thyroid hormones, parathyroid hormone (PTH), and other bone metabolism indicators, including serum osteocalcin (BGP), 25-hydroxyvitamin D (25(OH)D), and parathyroid hormone (PTH).

### Sarcopenia examination method

Participants were required to remove their shoes and socks before having their height measured. Participants were prohibited from carrying electronic devices. Next, the participants stood on predefined positions on the Biospace InBody720 or InBody770 body composition analyzer (InBody Co., Ltd., South Korea). Before administering the test, experienced nutritionists from the First Affiliated Hospital of Xinjiang Medical University collected the general information of the patients. During the test, participants remained still. The complete measurement process took roughly one minute. The completed results were then used to compute the Appendicular Skeletal Muscle Index (ASMI). The bioelectrical impedance analysis (BIA) method was applied to measure the appendicular skeletal mass (ASM) of the limbs and the whole body using the Biospace InBody720 Body Composition Analyzer or the InBody770 Body Composition Analyzer (InBody Co., Ltd.). The ASM was calculated as the sum of lean soft tissue from the arms and legs. ASMI was calculated as follows: ASMI = ASM/height^2^ (kg/m^2^) [18]. Based on the diagnostic criteria for sarcopenia, participants were subsequently categorized into the respective groups.

### Fatty liver examination method

Participants underwent abdominal ultrasonography (Canon Medical Systems, Canon, Japan) or hepatic ultrasound examinations that were conducted by experienced specialists from the ultrasound department of the First Affiliated Hospital of Xin Medical University. The results were determined based on the diagnostic criteria for fatty liver [7, 8]. 

### Diagnostic criteria

#### T2DM diagnostic criteria

All patients in this study were diagnosed with T2DM, i.e., they satisfied the WHO diagnostic criteria for diabetes established in 1999: possess typical diabetes symptoms of “three highs and one low” + random blood glucose level reaching or exceeding 11.1 mmol/L; fasting blood glucose reaching or exceeding 7.0 mmol/L; oral glucose tolerance test (OGTT) or 2-hour postprandial blood glucose measuring a blood glucose level that reaches or surpasses 11.1 mmol/L. For those patients without the typical “three highs and one low” symptoms or with only a single blood glucose measurement that reaches the diagnostic criteria, a re-check on a different day was required. The diagnosis was confirmed if the results were consistent with the criteria.

### Sarcopenia diagnostic criteria

Using the Bays’ InBody body composition analyzer (InBody Co., Ltd.), the BMI and muscle mass in the limbs and trunk of the participants were measured. The ASMI was subsequently calculated as a diagnostic indicator. The value for the sarcopenia diagnostic criteria was based on the lowest quintile of this indicator for a healthy young population of the same ethnicity and gender. The diagnostic values for sarcopenia are: for males ≤ 7.0 kg/m^2^, and for females ≤ 5.7 kg/m^2^.

### Fatty liver diagnostic criteria

The following were the diagnostic criteria for NAFLD [19] : (1) Enhanced diffuse echoes in the liver of the patient (i.e., “bright liver”), with these echoes being stronger than those from the kidney. (2) The anatomy and structures of the ducts in the liver are uncertain. (3) There is a diminishing tendency in the distant field for liver echoes. Diagnosis was confirmed if two of these criteria were met.

### Statistical analysis

The SPSS software (version 27.0, IBM, USA) was used for data processing and analysis. The normality of the obtained metric data was first tested using the Kolmogorov-Smirnov test method. Measurement data that fit the normal distribution were represented by mean ± standard deviation, and the independent samples *t*-test was used for comparison between the two groups. Measurement data that did not fit the normal distribution were represented by median and interquartile range, with the Mann-Whitney U test used for intergroup comparison analysis. Count data were represented by frequency and percentage, with comparison made using the chi-squared test. Factors affecting sarcopenia were analyzed using the multifactorial logistic regression method. The correlation between age, disease duration, BMI, and relevant laboratory markers with the ASMI was compared using Pearson’s correlation analysis. A difference was considered statistically significant if *P* < 0.05.

## Results

### Comparison of demographic data between the two groups

There were a total of 282 participants in this study (166 males and 116 females). Based on the relevant examination results and diagnostic criteria, the participants were divided into two groups: the sarcopenia group with 61 participants, representing 21.6% of the total, and the non-sarcopenia group with 221 participants, representing 78.4% of the total. As shown in Table 1, there were substantial differences between the two groups in terms of BMI, gender, and the incidence of fatty liver. Other markers, including disease duration, incidence of hypertension, smoking history, and alcohol drinking history, revealed no significant differences.

**Table 1:** Comparative analysis of the demographic data of patients having T2DM with and without sarcopenia

Item	T2DM without sarcopenia (*n* = 221)	T2DM with sarcopenia (*n* = 61)	P
Age (years)	63.23 ± 6.226	64.98 ± 7.683	0.092
Gender			< 0.001
Male	130(58.3%)	36(59.0%)	
Female	91(41.6%)	25(41.0%)	
Disease duration (years)	11.03 ± 7.78	13.6 ± 8.91	0.429
BMI	27.35 ± 3.889	24.019 ± 3.771	< 0.001
Hypertension	151(68.3%)	21(34.4%)	0.742
Smoking	49(22.2%)	17(27.8%)	0.527
Alcohol Consumption	40(18.1%)	11(18.0%)	0.817
Fatty liver	147(66.5%)	25(40.9%)	0.029

T2DM: type 2 diabetes mellitus; BMI: body mass index

Measurement data that fit the normal distribution were represented by mean ± standard deviation, while count data were represented by frequency and percentage

### Comparison of age, ASMI, and incidence of sarcopenia between patients diagnosed with T2DM with and without fatty liver

There were 176 patients diagnosed with T2DM with fatty liver and 106 without fatty liver. As indicated in Table 2, there were statistically significant differences in the two groups in terms of BMI and incidence of sarcopenia, but no significant differences in terms of ASMI and age.

**Table 2:** Comparative analysis of age, ASMI, and incidence of sarcopenia of patients with T2DM with and without fatty liver

Item	T2DM without fatty liver (*n* = 106)	T2DM with fatty liver (*n* = 176)	P
Age (years)	64.85 ± 6.723	63.43 ± 6.316	0.162
BMI(kg/m^2^)	24.25 ± 2.98	28.05 ± 2.96	< 0.001
ASMI(kg/m^2^)	7.586 ± 4.80	7.368 ± 0.955	0.658
Sarcopenia Incidence	28(26.4%)	19(10.8%)	0.029

T2DM: type 2 diabetes mellitus

Measurement data that fit the normal distribution were represented by mean ± standard deviation, while count data were represented by frequency and percentage

### Comparison of laboratory examination indicators between patients diagnosed with T2DM with and without sarcopenia

When comparing the relevant laboratory examination markers between patients diagnosed with T2DM having sarcopenia and those without, sarcopenia was associated with worse outcomes. According to Table 3, there were statistically significant differences in the levels of fasting c-peptide, TG, ALT, UA, and limb skeletal muscle content. Other than fasting c-peptide and TG, there were no statistically significant differences between the two groups in glucose metabolism, lipid metabolism, bone metabolism, AST, Cr, and albumin.

**Table 3:** Comparative analysis of laboratory examination data of patients with T2DM with and without sarcopenia

Item	T2DM without sarcopenia (*n* = 221)	T2DM with sarcopenia (*n* = 61)	P
FBG(mmol/L)	7.23 ± 2.76	6.69 ± 2.05	0.153
PBG(mmol/L)	15.30 ± 3.93	16.21 ± 4.97	0.280
FCP(mmol/L)	1.45(0.72,2.05)	1.25(0.56,1.62)	0.048
HbA1c(%)	8.60 ± 2.11	8.82 ± 2.19	0.461
TG(mmol/L)	1.38(0.94,2.26)	1.11(0.79,1.63)	0.028
TC(mmol/L)	4.08 ± 1.08	4.31 ± 1.22	0.147
HDL-C(mmol/L)	0.94(0.84,1.14)	1.03(0.88,1.26)	0.084
LDL-C(mmol/L)	2.64 ± 0.82	2.79 ± 0.94	0.179
ALT(U/L)	16.5(12.03,22.49)	13.27(9.52,17.52)	0.011
AST(U/L)	17.1(13.40,21.97)	15.2(12.6,21.9)	0.388
Cr(mmol/L)	75.63 ± 29.83	69.38 ± 19.24	0.114
UA(mmol/L)	305.62 ± 82.05	275.87 ± 68.99	0.009
Alb(g/L)	42.5(40.5,45.0)	41.3(39.50,44.80)	0.499
25(OH)D(nmol/l)	38.1(27.3,55.2)	46.6(29.31,56.37)	0.305
BGP(ng/ml)	12.87 ± 6.42	12.36 ± 4.89	0.551
PTH(pmol/l)	3.84 ± 1.57	3.62 ± 1.37	0.305
Ca(mmol/l)	2.25 ± 0.99	2.24 ± 0.11	0.449
ASMI(kg/m2)	7.63 ± 3.15	6.18 ± 0.68	< 0.001
Left Upper Limb	2.79 ± 0.66	2.08 ± 0.46	< 0.001
Right Upper Limb	2.79 ± 0.60	2.09 ± 0.45	< 0.001
Left Lower Limb	7.40 ± 1.37	5.98 ± 1.26	< 0.001
Right Lower Limb	7.43 ± 1.38	6.05 ± 1.26	< 0.001

T2DM: type 2 diabetes mellitus; FBG: fasting blood glucose; PBG: postprandial blood glucose; FCP: fasting c-peptide, HbA1c: glycated hemoglobin; TG: triglycerides; TC: total cholesterol; HDL-C: high-density lipoprotein cholesterol; LDL-C: low-density lipoprotein cholesterol; ALT: alanine aminotransferase; AST: aspartate aminotransferase; Cr: creatinine; UA: uric acid; Alb: albumin; 25(OH)D: 25-hydroxyvitamin D; BGP: serum osteocalcin; PTH: parathyroid hormone; Ca: serum calcium; ASMI: the Appendicular Skeletal Muscle Index.

Measurement data that fit the normal distribution were represented by mean ± standard deviation, while those that did not fit the normal distribution were represented by median and interquartile range. Count data were represented by frequency and percentage.

### Correlation between ASMI and various factors in patients with T2DM

According to Table 4, a positive correlation exists between BMI, UA, fasting c-peptide, and ASMI. On the contrary, there was no discernible association between ASMI and age, disease duration, TG, or ALT.

**Table 4:** Correlation between ASMI and other variables in patients with T2DM.

Factors	r	P
Age (years)	-0.059	0.413
Disease duration (years)	-0.061	0.397
BMI(kg/m^2^)	0.357	< 0.001
FCP(mmol/L)	0.162	0.036
TG(mmol/L)	0.047	0.527
ALT(U/L)	0.067	0.374
UA(mmol/L)	0.351	< 0.001

T2DM: type 2 diabetes mellitus; BMI: body mass index; FCP: fasting c-peptide; TG: triglycerides; ALT: alanine aminotransferase; UA: uric acid

### Multifactorial logistic regression analysis

The presence of sarcopenia was the dependent variable in this experiment. Age, gender, BMI, ASMI, FCP, TG, ALT, UA, and the existence of fatty liver were considered as independent variables. The logistic regression analysis revealed that BMI and ASMI may act as protective factors against sarcopenia in patients diagnosed with T2DM (as seen in Table 5).

**Table 5:** Multivariate logistic regression analysis of sarcopenia in patients with T2DM with factors such as fatty liver, BMI, and so on

Factors	β	SE	Wald	P	OR	95%CI
Age (years)	0.029	0.038	0.588	0.443	1.057	0.980 ~ 1.140
Gender	-7.814	1.376	32.252	0.557	0.735	0.262 ~ 2.058
BMI(kg/m^2^)	-0.383	0.090	18.118	< 0.01	0.682	0.571 ~ 0.913
ASMI(kg/m^2^)	-1.845	0.342	29.154	< 0.01	0.158	0.081 ~ 0.309
FCP(mmol/L)	0.364	0.238	2.347	0.126	1.440	0.903 ~ 2.295
TG(mmol/L)	-0.235	0.217	1.173	0.279	0.791	0.517 ~ 1.209
ALT(U/L)	-0.011	0.031	0.115	0.734	0.990	0.931 ~ 1.052
UA(mmol/L)	-0.008	0.004	3.638	0.056	0.992	0.984 ~ 1.000
Fatty liver	0.846	0.467	3.288	0.07	2.331	0.934 ~ 5.817

T2DM: type 2 diabetes mellitus; BMI: body mass index; ASMI: the Appendicular Skeletal Muscle Index; FCP: fasting c-peptide; TG: triglycerides; ALT: alanine aminotransferase; UA: uric acid; OR: odds ratio; CI: confidence interval

## Discussion

### Current state of diabetes and its complications in Middle-aged and elderly individuals

Diabetes, being a chronic disease that can affect the overall metabolic status of the body, can result in a range of macrovascular to microvascular complications if it is not addressed properly [20, 21]. Long-term failure to control blood glucose levels efficiently can result in a variety of issues. Some of these problems are even life-threatening [22]. 

A meta-analysis of 1 832,125 patients identified the prevalence of Non-alcoholic fatty liver disease (NAFLD) in T2DM as 65.04%, respectively [23]. The frequency of fatty liver in populations with normal blood glucose levels ranges between 20 and 30%. However, in patients diagnosed with T2DM, the prevalence of this condition may increase to 70–80% [24, 25]. In this study, roughly 62.4% of patients had NAFLD, which is consistent with other studies [24, 25]. Patients with fatty liver have greater blood glucose levels than those with a healthy liver [26]. A meta-analysis of 1,832,125 patients believed that the prevalence of NAFLD in T2DM was 65.04% [27]. 

Sarcopenia is a frequent complication in middle-aged and elderly patients diagnosed with T2DM. Approximately 4.1–11.5% of Asian middle-aged and elderly populations suffer from sarcopenia [28]. Patients diagnosed with sarcopenia undergo more rapid bone degeneration and are at a greater risk for osteoporosis than the general population. This increases the probability of falls and fractures, which has a substantial effect on daily life, and could result in debilitating conditions or even death in more extreme cases [16, 17]. Regarding treatment, no specific medications are currently available. Interventions for muscle atrophy primarily include resistance training, blood flow restrictive training, nutritional supplementation, and medication. Currently, resistance training is widely recognized as the most effective intervention for treating muscle atrophy or reducing its progression. Another major treatment approach for muscle atrophy is nutritional intervention, which involves supplementing with protein, vitamin D, leucine, etc., typically in conjunction with the aforementioned exercise training programs [29]. Currently, there are no specific medications approved for treating muscle atrophy [30]. Consequently, determining the origins and triggers of sarcopenia is crucial for its prevention and control.

#### Relationship between gender and sarcopenia

Due to the fundamental variances between male and female endocrine systems, there are also discrepancies in their metabolic rates. The results of this study imply that gender influences the onset of sarcopenia. Notably, the incidence of sarcopenia is substantially higher in men than in women. This disparity may be explained by the fact that males and females have different hormone levels. In addition, as people age, male and female hormonal swings diverge. Androgens, or male hormones, play a crucial function in muscle protein synthesis. Consequently, a fall in testosterone levels eventually results in a loss of muscle mass [31]. The pathogenesis of muscle atrophy may be related to factors such as aging, chronic inflammation, reduced hormone levels, mitochondrial dysfunction, nutritional deficiency, and lack of physical activity, leading to a decrease in motor neurons and satellite cell numbers, and muscle loss [32]. Some hormones, including testosterone, play a role in regulating protein synthesis and breakdown processes in muscles. Aging or other factors leading to a decline in hormone levels can result in reduced muscle synthesis metabolism, leading to loss of muscle mass and strength [33]. The impact of gender on the prevalence of muscle atrophy varies in research, with inconsistent results. A systematic review and meta-analysis suggest an overall prevalence of around 10% for community-dwelling elderly individuals with muscle atrophy, with rates of 11% for males and 9% for females. In hospitalized individuals, the rates are 23% for males and 24% for females, and in nursing homes, the rates are 51% for males and 31% for females [34]. Another study indicates a higher prevalence of muscle atrophy in non-Asian populations compared to Asian populations (males: 11% vs. 10%; females: 12% vs. 9%) [35]. Different diagnostic methods and equipment may account for the disparate results obtained by different investigations. Moreover, due to the dynamic nature of hormone levels within the human body, potential errors may occur.

This study did not identify gender as an independent risk factor for sarcopenia, which was similar to a study from Japan [36]. 

### Association between glucose metabolism and sarcopenia

T2DM has a genetic susceptibility, with potentially thousands of genetic factors contributing to disease risk, interacting in complex ways with environmental factors [37]. The mechanisms by which glucolipotoxicity affects pancreatic function have been increasingly studied this year, indicating that the glucose toxicity mechanism in pancreatic β-cells is highly complex, involving multiple aspects and pathways. Currently, oxidative stress is considered to play a crucial role in the glucotoxicity of pancreatic cells in diabetes [38]. The roles of endoplasmic reticulum stress and the loss of pancreatic β-cell differentiation phenotype in the glucotoxicity of pancreatic β-cells are relatively clear [38, 39]. Reduced insulin sensitivity is the primary cause of T2DM. Based on contemporary research [40], skeletal muscle is the largest insulin target organ in the body. Consequently, diminished insulin sensitivity not only has substantial consequences for blood glucose homeostasis, but also has a considerable effect on skeletal muscles, a situation that may prompt the onset of sarcopenia. Insulin resistance can cause hyperglycemia and an increase in insulin levels throughout the body. In these conditions, the ability of muscle cells to produce proteins is impaired. T2DM can also cause disruptions or even anomalies in mitochondrial activity, hence increasing insulin resistance and speeding the evolution of sarcopenia. According to Stephen et al., patients without sarcopenia have lower HbA1c levels than those with sarcopenia based on blood glucose and skeletal muscle index assessment [41]. Currently, the pathophysiological mechanisms of diabetic myopathy remain a widely debated topic. Widely recognized possible causes include ischemia [42], impaired mitochondrial function [43], and inflammation [44], among other factors. Specific treatments are still focused on glycemic control [45]. Acupuncture support therapy lacks specific treatment methods, so prevention is emphasized [46]. Other potential contributing variables may include insulin secretion deficiencies, mitochondrial damage, systemic metabolic abnormalities, persistent inflammation, and other diabetes-related problems [47]. 

#### Associations between other metabolic indicators and sarcopenia

##### Blood lipids

The modulatory effects of fatty acids and their metabolic intermediates on skeletal muscle function are supported by recent studies. A study by Gong et al. included 84 patients aged 65 and above [48]. Blood lipid measurements, the SMI, and grip strength were assessed in the study. Patients with sarcopenia had significantly higher levels of TC, TG, LDL-C, and very low-density lipoprotein than the general population or the control group. Through Pearson’s correlation analysis, it was determined that an increase in blood lipid levels accompanied a decline in muscle strength, demonstrating a negative association between the two. Wang et al. investigated the skeletal muscle mass and blood lipid levels of 2,613 patients in another study [49]. In all, 13.85% of the patients were affected by sarcopenia. The results indicated a negative correlation between the onset of sarcopenia and TG and a favorable correlation with HDL-C. In addition, patients without sarcopenia had higher average blood lipid levels than those with sarcopenia.

#### UA

The relationship between UA and various diseases is complex due to the dual nature of UA, which includes both oxidative and antioxidant properties. Gout, renal disease, metabolic disorders, and obesity may be precipitated by elevated UA levels in the human body. Despite this, because of its antioxidant properties, UA is essential for maintaining normal physiological functions [50, 51]. Currently, there are few studies that have investigated the relationship between sarcopenia and UA, and their results are highly variable. Based on research conducted by Beaver et al., elevated UA may lead to the onset of sarcopenia [52]. Blood UA levels that are too high can induce systemic inflammatory reactions and increased oxidative stress, hence increasing the risk of sarcopenia.

Another study indicated a negative correlation between serum UA levels and sarcopenia, irrespective of gender [53]. In contrast, UA levels were positively correlated with skeletal muscle index and grip strength. This study revealed a significant correlation between elevated UA levels and enhanced muscle hypertrophy and grip strength. Potentially, elevated serum UA levels may delay the progression of sarcopenia. Although an excessively high UA level can lead to a variety of disorders, its vital function in everyday life cannot be neglected or denied. The incidence of sarcopenia is closely associated with UA. Whether blood UA functions as a risk factor or a protective factor for sarcopenia is yet unclear. Further research with bigger sample sizes are required to rule out other confounding variables and investigate this association in greater depth.

### Association between fatty liver and sarcopenia

The risk of developing sarcopenia and fatty liver is increased by T2DM. Based on previous studies [54, 55] involving patients with normal blood glucose levels, individuals with sarcopenia are at a greater risk for developing fatty liver [56, 57]. The results of a meta-analysis that assessed 1,331 relevant publications and statistically analyzed 19 of them suggested that the muscle mass of fatty liver patients was lower than that of the control group, indicating an increased risk of fatty liver in this demographic [58]. The outcomes of this study indicate that sarcopenia is strongly negatively correlated with BMI in elderly patients diagnosed with T2DM. The non-sarcopenic group revealed a greater incidence of fatty liver as compared to the sarcopenic group. However, based on logistic regression analysis, fatty liver is neither a risk factor nor a protective factor for sarcopenia. The study by Sun et al. [59] found that higher levels of UA appear to have a protective effect against sarcopenia in male participants in a study on elderly patients with T2DM aged 65 and above. In our study, we did not separately analyze participants of different genders. Instead, gender was considered as one of the independent variables in the multifactorial analysis, along with factors such as age and fasting C-peptide levels. The analysis did not reveal a protective effect of UA against sarcopenia in the participants. The correlation between uric acid and sarcopenia in different gender groups needs further exploration. Additionally, similar to the mentioned study, our study also showed a protective effect of BMI against sarcopenia.

The strength of this article lies in the consideration of indicators affecting bone metabolism, such as parathyroid hormones, blood calcium levels, and 25-hydroxyvitamin D, when designing the study population. This enriched the exploration of potential factors linking sarcopenia and fatty liver in middle-aged and elderly patients with T2DM. The study suggests that higher levels of BMI and ASMI can reduce the occurrence of sarcopenia, providing more clues for understanding the potential correlation between fatty liver and sarcopenia. However, there are some limitations to this study. Firstly, the number of subjects included in the study is relatively small, and the proportion of sarcopenia among the subjects is low. Secondly, all enrolled patients in this study were hospitalized in the endocrinology department, raising the possibility of selection bias. Additionally, the diagnosis of fatty liver relied solely on liver ultrasound examination, without further assessments such as liver fibrosis evaluation or biopsy, thus requiring further validation of the conclusions.

## Conclusion

In conclusion, our investigation into hospitalized patients within the endocrinology department, following rigorous screening, thorough testing, and careful exclusion of confounding variables, has yielded data-driven insights. Significant differences were observed in gender composition, BMI, and the incidence of fatty liver between sarcopenic and non-sarcopenic groups. Furthermore, marked variations in fasting c-peptide, TG, ALT, UA, and limb skeletal muscle content were identified when comparing the two groups. Our findings suggest a positive correlation between BMI, UA, fasting c-peptide, and ASMI levels. Moreover, through multivariate logistic regression analysis, we observed that higher BMI and ASMI serve as protective factors against sarcopenia. These findings may contribute valuable insights to the understanding of the relationships between various factors and sarcopenia.

## Data Availability

The data used to support the findings of this study are available from the corresponding author upon request.
